# Effects of Hydrophilic Carriers on Structural Transitions and In Vitro Properties of Solid Self-Microemulsifying Drug Delivery Systems

**DOI:** 10.3390/pharmaceutics11060267

**Published:** 2019-06-08

**Authors:** Tao Yi, Jifen Zhang

**Affiliations:** 1 School of Health Sciences, Macao Polytechnic Institute, Macao 999078, China; yitao@ipm.edu.mo; 2 College of Pharmaceutical Sciences, Southwest University, Chongqing 400716, China

**Keywords:** solid self-microemulsifying drug delivery systems, hydrophilic carriers, microstructure, dissolution, in vitro methods

## Abstract

Self-microemulsifying drug delivery systems (SMEDDS) offer potential for improving the oral bioavailability of poorly water-soluble drugs. However, their susceptibilities during long term storage and in vivo precipitation issues limit their successful commercial application. To overcome these limitations, SMEDDS can be solidified with solid carriers, thus producing solid self-microemulsifying drug delivery systems (S-SMEDDS). In this study, effects of various hydrophilic carriers on structural transitions and in vitro properties of S-SMEDDS were investigated in order to set up in vitro methods for screening out appropriate carriers for S-SMEDDS. Liquid SMEDDS was prepared and characterized using nimodipine as a model drug. The effects of various hydrophilic carriers on internal microstructure and solubilization of SMEDDS were investigated by conductivity measurement and in vitro dispersion test. The results showed that hydrophilic carriers including dextran 40, maltodextrin and PVP K30 seemed to delay the percolation transition of SMEDDS, allowing it to maintain a microstructure that was more conducive to drug dissolution, thus significantly increasing the solubilization of nimodipine in the self-microemulsifying system and decreasing drug precipitation when dispersed in simulated gastric fluid. S-SMEDDS of nimodipine were prepared by using spray drying with hydrophilic carriers. The effects of various hydrophilic carriers on in vitro properties of S-SMEDDS were investigated by using SEM, DSC, PXRD and in vitro dissolution. The results showed that properties of hydrophilic carriers, especially relative molecular mass of carriers, had obvious influences on surface morphologies of S-SMEDDS, reconstitution of microemulsion and physical state of nimodipine in S-SMEDDS. Considering that in vitro properties of S-SMEDDS are closely related to their pharmacokinetic properties in vivo, the simple and economical in vitro evaluation methods established in this paper can be used to screen solid carriers of S-SMEDDS well.

## 1. Introduction

Oral administration is one of the most simple, noninvasive and acceptable medication routes for most patients. However, the oral bioavailabilities of poorly soluble and/or poorly permeable drugs have been extremely low, which limits their clinical use by oral administration. For example, nimodipine is a dihydropyridine calcium channel blocker that is clinically used in preventing a major complication of subarachnoid hemorrhage. However, its absolute bioavailability after oral administration is as low as about 13%, thus resulting in an extraordinarily high required dose of about 360 mg per day [1]. Therefore, development of new formulations for improving oral absorption of poorly soluble and/or poorly permeable drugs has been a sustained focus of pharmaceutics. Many lipid formulations, such as solid lipid nanoparticles (SLN) [2], nanostructured lipid carrier (NLC) [3], and nanoemulsions [4] have been developed to improve the oral bioavailability of nimodipine. Among various strategies, the self-microemulsifying drug delivery system (SMEDDS) has attracted much attention. SMEDDS is an isotropic mixture with drugs dissolved or suspended in a mixture of oils, surfactants, and hydrophilic co-solvents, which can form spontaneously oil-in-water microemulsion in aqueous media under mild digestive motility of the gastrointestinal tract (GIT) [5]. It has been widely proven that SMEDDS is one of the most effective approaches to improve drug solubility and dissolution, and oral absorption of poorly water-soluble drugs [6,7,8]. However, there are also some shortcomings for SMEDDS, such as the risk of GIT irritation caused by a relatively high proportion of hydrophilic surfactants (20%–50%) and co-solvents (20%–50%) in SMEDDS, physical destabilization of the in situ formed microemulsions, drug crystallization and precipitation in vivo which becomes unavailable for absorption due to dispersion of gastric liquid and/or lipolysis digestion of small intestine lipase [9,10]. In addition, just like SLN, NLC and nanoemulsions, SMEDDS also is a liquid form and is inconvenient for transportation and clinical applications.

Solid dosage form is preferable because of its good physicochemical stability, convenience of manufacturing, patient compliance and cost-performance. Therefore, transforming SMEDDS into a solid dosage form became a promising approach to overcome its fundamental drawbacks while retaining its pharmacokinetic benefits [11,12]. Various solid self-microemulsifying drug delivery systems (S-SMEDDS) have been investigated by adding solid carriers to solidify SMEDDS [13,14,15], such as silica-based water-insoluble adsorbents (e.g., porous silica), cellulose-based hydrophilic diluents (e.g., microcrystalline cellulose, hydroxypropyl methyl cellulose) and saccharide-based water-soluble diluents (e.g., maltodextrin, lactose) [16,17,18].

Good S-SMEDDS must keep all the inherent merits of liquid SMEDDS. Appropriate solid carriers for S-SMEDDS could be selected by comparing the pharmacokinetic properties in vivo between S-SMEDDS and SMEDDS [19]. In our previous study, the oral bioavailability in rabbits demonstrated that S-SMEDDS loading nimodipine (S-SMEDDS-Ni) with dextran as the solid carrier could preserve an improved bioavailability with releasing microemulsion droplets from the formulation in vivo [20]. However, the mechanism of such a property of the solid carrier is not clear. Determining the influences of solid carrier on in vitro properties of S-SMEDDS, especially the structural transitions of reconstructed microemulsions after redispersed in water and drug loading, as well as precipitation and dissolution of S-SMEDDS in simulated gastric fluid, is essential for the reasonable choice of solid carriers. In addition, pharmacokinetic study in vivo was labor-intensive and expensive. Since characteristics in vitro of S-SMEEDS were closely related to their pharmacokinetic properties in vivo [21,22], it was considered a reasonable, economical and convenient method to select proper solid carriers by studying influences of solid carriers on properties of S-SMEDDS with in vitro experiments.

In this study, we tried to compare the influences of different hydrophilic carriers on in vitro properties of S-SMEDDS-Ni and thereby, set up good in vitro methods to optimize a suitable carrier for SMEDDS. Influences of various hydrophilic carriers on in vitro characteristics of SMEEDS, including microstructural transitions, droplet size, drug loading, dispersion and precipitation of SMEDDS in simulated gastric fluid, were systematically studied. The effects of hydrophilic carriers on in vitro properties of S-SMEEDS, such as micromorphology, reconstruction of microemulsion, physical state of nimodipine and dissolution, were also assessed.

## 2. Materials and Methods

### 2.1. Chemicals and Reagents

Nimodipine (purity > 99.5%) and nimodipine tablet were purchased from Kaifeng Pharmaceutical (Group) Co., Ltd. (Kaifeng, China). Ethyl oleate was purchased from Shanghai Chemical Reagent Factory (Shanghai, China). Labrasol^®^ and Cremophor^®^ RH 40 were purchased from Gattefossé Corp., Lyon, France and BASF Corp., Lampertheim, Germany, respectively. Dextran 40 of pharmaceutical grade (weight-average molecular weight of 40,000) was purchased from Shanghai Huamao Pharmaceutical Co., Ltd. (Shanghai, China). Maltodextrin of medicinal grade was purchased from Shanghai Yun Hong Chemical Co., Ltd. (Shanghai, China). PVP K30 of pharmaceutical grade was purchased from Shanghai Pharmaceutical Excipient Factory (Shanghai, China). Acacia of analytical grade was purchased from Sigma Chemical Co. (St. Louis, MO, USA). Other solvents and chemicals were of analytical grade.

### 2.2. Preparation of SMEDDS and Droplet Size Determination

SMEDDS-Ni was prepared based on our pre-experiment and literature [23]. Briefly, 280 mg of Cremophor^®^ RH, 7 mg of Labrasol^®^ and 5 mg of nimodipine were mixed at 37 °C until nimodipine was dissolved completely. Then 600 mg of ethyl oleate was added and shaken slowly at 37 °C to obtain a transparent and homogeneous liquid. Blank SMEDDS was prepared using the same procedure as SMEDDS-Ni without nimodipine being added.

The droplet size determination was carried out as follows. SMEDDS of 50 μL was added to pure water of 10 mL and vortex-mixed for 30 s. After standing for 30 min at 25 °C, the droplet size of resultant microemulsion was measured by photon correlation spectroscopy (PCS) at a wavelength of 635.0 nm, a scattering angle of 90° and a temperature of 25 °C with a Nano series ZS instrument (Zetasizer Nano-ZS, Malvern Instruments, Malvern, UK).

### 2.3. Effects of Carriers on Microstructure of SMEDDS

A series of microemulsions with water content varying from 0 to 95% were obtained by adding different amount of water into blank SMEDDS or SMEDDS-Ni. The conductivities of resultant microemulsions were measured with a DDS-2A conductivity meter (Shanghai Second Analytical Instrument Factory, Shanghai, China) and the conductivity-water content curves were drawn. The viscosities of resultant microemulsions near percolation thresholds were also measured by NDJ-8S digital viscometer (Shanghai Jingtian Electronic Instrument Co., Ltd., Shanghai, China). In the same way, different hydrophilic carrier solutions (5%, *w*/*v*) were added to SMEDDS-Ni, respectively, and the conductivity-water content curves were measured using the aforementioned method.

### 2.4. Effects of Carriers on Drug Loading of SMEDDS

Excessive nimodipine was added into a series of SMEDDS with water content of 0–90%. The mixture was vortex-mixed for 1 min and then shaken in the dark at 37 °C for 72 h. Finally, the mixture was centrifugated at 6000 rpm for 5 min. Nimodipine concentrations in supernatants were determined by high performance liquid chromatography (HPLC) and solubilities of nimodipine in SMEDDS with different water content were calculated. The solubilities of nimodipine in mixtures of SMEDDS and hydrophilic carriers were also measured in the same way.

HPLC analysis of nimodipine was conducted in an Agilent 1100 system with a Lichrospher C18 column (4.6 mm × 250 mm, 5 μm particle size). The mobile phase consisted of 0.05 mol·L^−1^ ammonium acetate and acetonitrile (35: 65, *v*/*v*). The flow rate was set to 1.0 mL·min^−1^ and column temperature was set to 30 °C. The detection wavelength was 237 nm [20]. The HPLC method was verified according to the Chinese Pharmacopoeia (2015 edition). The retention time of nimodipine was 7.6 min and excipients in formulations did not affect determination of nimodipine. The linear range was 3.00–300.00 μg·mL^−1^ (*r* = 0.9999). The intra-day and inter-day precision were 1.52% and 2.30%, respectively. The RSD of the repeatability test was 2.83% and the accuracy was 98.72%.

### 2.5. Effects of Carriers on Dispersion and Precipitation of SMEDDS in Simulated Gastric Fluid

SMEDDS-Ni of 2g was added into simulated gastric fluid (0.1 mol·L^−1^ HCl) of 200 g and stirred at 100 rpm, 25 °C in the dark. Samples were withdrawn at 0, 5,15 min, 24, 48, 72, 96, 120 and 144 h and centrifugated at 6000 rpm for 5 min. Nimodipine concentrations in supernatants were determined by HPLC mentioned above and amounts of dissolved nimodipine were calculated. In the same way, the amounts of nimodipine dissolved in simulated gastric fluid containing hydrophilic carriers (1%, *w*/*v*) were also measured.

### 2.6. Preparation of S-SMEDDS

S-SMEDDS was prepared based on preliminary experiments. Hydrophilic carrier of 10.0 g was dispersed in pure water of 100 mL and stirred until dissolved completely. Subsequently, SMEDDS-Ni of 10.0 g was added and stirred for 10 min. The resultant mixture was spray-dried using a B-191 Mini Spray-dryer (Büchi, Flawil, Switzerland), employing a flow rate of 5 mL·min^−1^, dry air flow rate of 500 NL·h^−1^, inlet temperature of 120 °C, which resulted in an outlet temperature of 70 °C.

### 2.7. Morphological Analysis of S-SMEDDS

The morphologies of S-SMEDDS were assessed by scanning electron microscopy (SEM). Samples were placed on a double-side electro-conductive adhesive tape which was fixed on an aluminum stub, and then sputter-coated with gold under argon atmosphere. SEM micrographs were taken using a FEI Sirion-200 SEM (Thermo Fisher Scientific Inc., Bleiswijk, The Netherlands).

### 2.8. Reconstitution Properties of S-SMEDDS

SMEDDS-Ni of 50 μL and S-SMEDDS-Ni of 100 mg prepared with different carriers were respectively diluted with 10 mL pure water and then were shaken vigorously for 30 s. After setting quietly for 30 min, droplet sizes of resultant microemulsions were measured.

### 2.9. Characterization of Inner Physical Structure of S-SMEDDS

Nimodipine raw material, S-SMEDDS-Ni and mixtures of nimodipine with different carriers were analyzed by differential scanning calorimetry (DSC) and powder X-ray diffraction (PXRD). Accurately weighted samples of 5 mg were placed in open aluminum pan. DSC was performed on a diamond differential scanning calorimeter (PerkinElmer, Waltham, MA, USA) at 5 °C·min^−1^ in the range of 10–150 °C under a nitrogen purge gas flow of 40 mL·min^−1^. PXRD was carried out with an X’Pert PRO diffractometer (PANalytical Inc., Almelo, Netherland). Cu Ka radiation at 40 mA and 40 kV with a step of 0.02° and a speed of 2° (2θ)·min^−1^ were used, covering a 2θ range of 10–40°.

### 2.10. In Vitro Dissolution Studies of S-SMEDDS

The dissolution of S-SMEDDS-Ni and nimodipine tablets were studied using Chinese Pharmacopoeia II apparatus with paddles [24]. Acetate buffer of 900 mL with pH of 4.5 containing sodium lauryl sulfate (0.05%, *w*/*v*) was used as the dissolution medium. Equivalent amounts of S-SMEDDS-Ni and nimodipine tablets (containing 10 mg of nimodipine) were put into the dissolution medium of 37 °C and stirred at 75 rpm, respectively. Samples of 2 mL were collected at designed intervals and equivalent fresh media were added. The collected samples were filtered through a millipore filter of 0.22 μm and drug concentrations were quantified by the HPLC method mentioned above.

### 2.11. Statistical Analyses

All data were expressed as mean ± standard deviation (S.D.). One-way ANOVA was used to test the differences between groups and *P* < 0.05 or *P* < 0.01 was considered to be a significant difference.

## 3. Results and Discussions

### 3.1. Effects of Carriers on Microstructure of SMEDDS

Conductivity was commonly used to characterize microstructures of microemulsions [25]. The percolation threshold was determined from the plot (dκ/dw), as a function of the water weight fraction, which was the maximum in the first derivative [26]. The percolation threshold indicated that a percolation phase transition occurred in SMEDDS, i.e., a transition from a W/O microemulsion to a bi-continuous phase structure [27,28].

**Figure 1:**
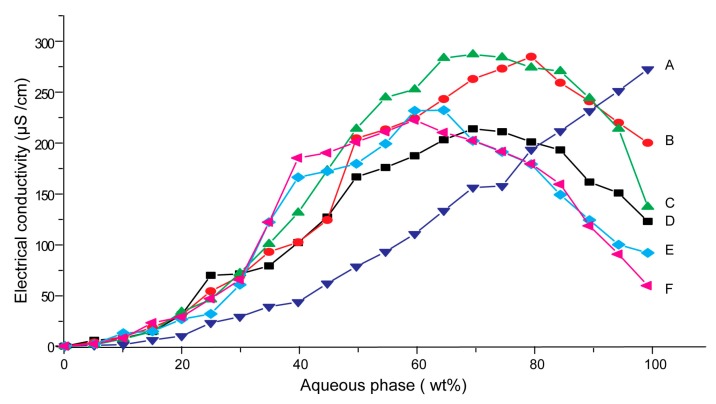
Electrical conductivity as a function of the water content (wt. %) for self-microemulsifying drug delivery systems (SMEDDS) containing (**A**) acacia, (**B**) dextran 40, (**C**) maltodetrin, (**D**) PVP K30, or (**E**) blank microemulsion and (**F**) drug loaded microemulsion.

Figure 1 showed the electrical conductivity as a function of water content for blank SMEDDS and SMEDDS-Ni, as well as mixtures of SMEDDS-Ni and different carriers. The curves of blank SMEDDS and SMEDDS-Ni were similar (Figure 1A), and their percolation thresholds both were 35%, which suggested that nimodipine had no influence on the microstructure of SMEDDS.

The electrical conductivity–water content curves for mixtures of SMEDDS and dextran 40, maltodextrin or PVP K30, respectively, were all basically similar to SMEDDS-Ni (Figure 1B–D). The curve could be divided into three stages, which corresponded to three microstructures of SMEDDS, respectively. At first, the electrical conductivity increased slowly with increasing of water content from 0 to 20%. This may be because the system was the W/O microemulsion, which had few charged emulsion droplets. Then, the electrical conductivity varied according to a bell-shaped curve with a peak at about 70 wt. % of water content. This indicated that the system changed to interconnected bi-continuous structure [29]. At last, when water content of microemulsion was more than 80 wt. %, its conductivity decreased rapidly. The reason may be that the system had changed to the O/W microemulsion and the viscosity of system increased rapidly with the increasing of the water content, which leaded to a decrease of electrical conductivity [27,29]. Microstructural changes were closely related to drug loading capacity, which would be discussed in detail later. The addition of the three carriers did not change the variation tendency of SMEDDS microstructure, which implied that S-SMEDDS with them as carriers might maintain the solubilizing ability of SMEDDS. The percolation threshold of microemulsions all increased from 35% to 45% when hydrophilic carriers were added into SMEDDS-Ni, respectively, which suggested that hydrophilic carriers hindered percolation phase transition of SMEDDS. On the one hand, hydrophilic carriers increased viscosities of emulsion systems. The viscosities of resultant microemulsions with acacia, maltodextrin, PVP K30 and dextran as solid carriers at 35% (wt. %) of water content were 4656.2 ± 64.8, 3232.7 ± 85.5, 8048.1 ± 90.0, 3806.7 ± 91.1 mPa·s, respectively, which were all higher than 3006.1 ± 38.0 of the control group. The viscosities at 50% (wt. %) of water content also had the same trend. The viscosities for blank, acacia, maltodextrin, PVP K30 and dextran group were 421.3 ± 5.3, 565.4 ± 12.1, 468.2 ± 2.5, 3417.3 ± 23.4 and 644.3 ± 14.2 mPa·s, respectively. The conductivity can decrease as the viscosity increases [30]. The addition of the carriers reduced the rate of change in the conductivity of the systems, thereby increasing the percolation threshold. On the other hand, carriers could form a protective film at the oil-water interface, which could hinder the interconnection between the droplets and increase the stability of the emulsion droplets, thus delaying the percolation phase transition. The delay in phase transition meant that the solubilization capacity could be maintained for a longer period of time. Therefore, the above results suggested that the three carriers might be beneficial for solubilization of SMEDDS.

However, acacia had a different effect on SMEDDS-Ni from dextran 40, maltodextrin and PVP K30. The electrical conductivity of the mixture of SMEDDS-Ni and acacia always increased with increasing of water content (Figure 1E). This may be due to plentiful electrolytes in acacia, which made charged emulsion droplets continue to increase as the water content increased, resulting in a continuous rise in conductivity.

### 3.2. Influences of Carriers on Drug Loading of SMEDDS

**Figure 2:**
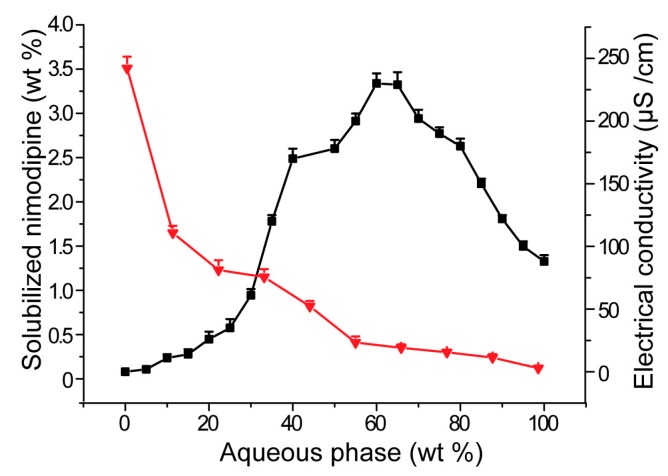
Nimodipine solubilization capacity (▼) and the electrical conductivity (⬛) of SMEDDS upon dilution with water (*n* = 3).

The microstructure of SMEDDS is closely related to the drug loading capacity and drug release rate [31]. The state of the drug, i.e., whether it is precipitated, and the drug loading capacity of the system are important performance indicators of SMEDDS. Therefore, it is important to study the drug loading capacity of the system with the change of microstructure. In this study, electrical conductivity change of SMEDDS was used as an indication of microstructure. Like the electrical conductivity–water content curve, the drug loading-water content curve for SMEDDS could also be divided into three regions (Figure 2). In the first region of W/O microemulsion with water content between 0 and 20%, the drug loading decreased rapidly with increasing of water content. The reason may be that, after adding small amount of water, the initial reverse micelle structure in the absence of water had changed to W/O microemulsion [15]. It was well known that reverse micelles had a higher drug loading than W/O microemulsion. Therefore, the drug-loading capacity of the system rapidly decreased in the first stage. Secondly, the decreasing rate of drug loading capacity of SMEDDS slowed down from a water content of about 30%. The reason for this may be that the system began to form a bi-continuous structure in this region and the drug migrated from the oil-rich region to the oil-water interface. With increasing of the water content, the oil-water interface decreased relatively slowly, which led to a moderate decline of drug loading. At the last stage, when water content was up to 50%, drug loading of SMEDDS decreased much more slowly and was nearly linear with water content. The slow decrease of drug loading may be due to the fact that the amount of dissolved drug in the system was very small. The linear relationship may be due to the fact that the drug loading capacity of SMEDDS in the last region mainly derived from hydrophilic surfactants, and drug solubility was generally linear with the concentration of surfactants [32].

**Figure 3:**
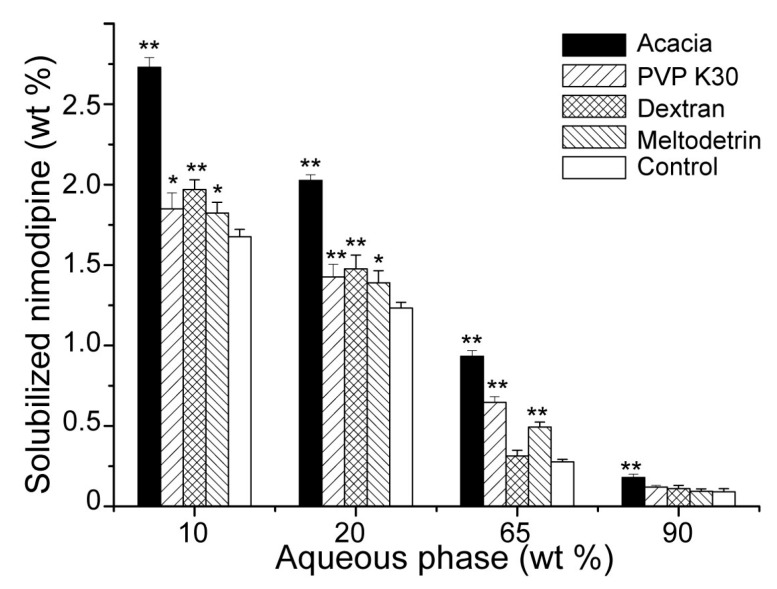
Effects of hydrophilic carriers on solubilization capacity of SMEDDS to nimodipine upon dilution with water. The control group was the SMEDDS without any carriers (*n* = 3). ^*^P < 0.05, ^**^ *P* < 0.01 vs. control group.

The effects of carriers on drug loading capacity of SMEDDS were shown in Figure 3. Compared with SMEDDS-Ni without any carriers, addition of acacia, PVP K30, dextran and maltodextrin all enhanced drug loading of SMEDDS-Ni when water content was lower than 65%. This was consistent with the effects of carriers on the SMEDDS microstructure. As shown in Figure 2, hydrophilic carriers delayed the phase transition of SMEDDS and SMEDDS maintained a microstructure with higher solubility when the water content increased, which increased the drug loading of SMEDDS. Acacia had the strongest solubilization ability and maintained a higher drug loading than the control group even if water content was up to 90%. This may be due to the partially emulsifying ability of acacia itself.

### 3.3. Influences of Carriers on Dispersion and Precipitation of SMEDDS in Simulated Gastric Fluid

**Figure 4:**
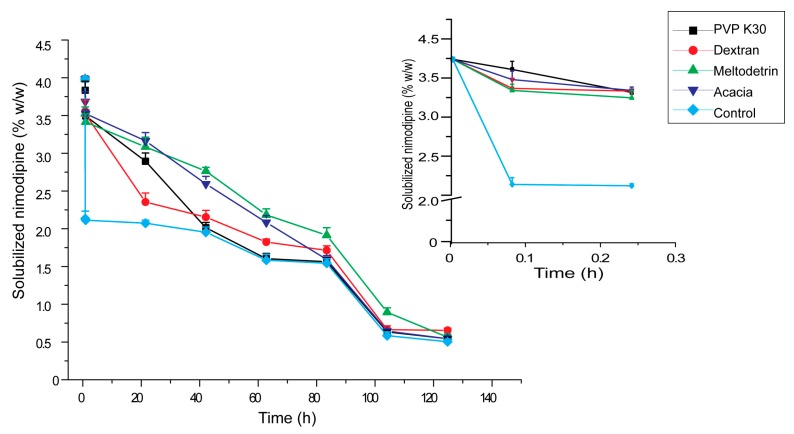
Effects of hydrophilic carriers on the in vitro dispersion of SMEDDS in simulated gastric fluid. The control group was the SMEDDS without any carriers (*n* = 3).

The evaluation of lipid preparations in vitro mainly focused on the rate and extent of drug precipitation. Dispersion experiment was used to investigate the ability of lipid preparations to maintain drugs in a dissolved state when they were dispersed in simulated gastric fluid [33,34]. The results were shown in Figure 4. After being dispersed in simulated gastric fluid, approximately half of nimodipine precipitated quickly from SMEDDS-Ni without carriers. This occured because that SMEDDS contained a large amount of water-miscible surfactants or co-solvents, and as mentioned above, drug solubility of SMEDDS in the region of high water content was more dependent on the concentrations of surfactants and co-solvents. By contrast, it took about 100 h for SMEDDS-Ni with acacia, PVP K30, dextran and maltodextrin as carriers to precipitate about half of nimodipine, indicating that these four carriers all could inhibit drug precipitation during dispersion of SMEDDS in vitro and enhance the ability of SMEDDS to maintain drugs in a dissolved state. This was consistent with the results of the above studies on drug loading and microstructure of SMEDDS. The hydrophilic carriers could hinder the microstructure transformation of SMEDDS and increase the drug loading. Therefore, a similar supersaturation state would be kept when SMEDDS was dispersed in simulated gastric liquid, which could reduce drug precipitation [35,36]. This was beneficial for oral absorption of SMEDDS.

### 3.4. Influences of Carriers on Characterization of S-SMEDDS

**Figure 5:**
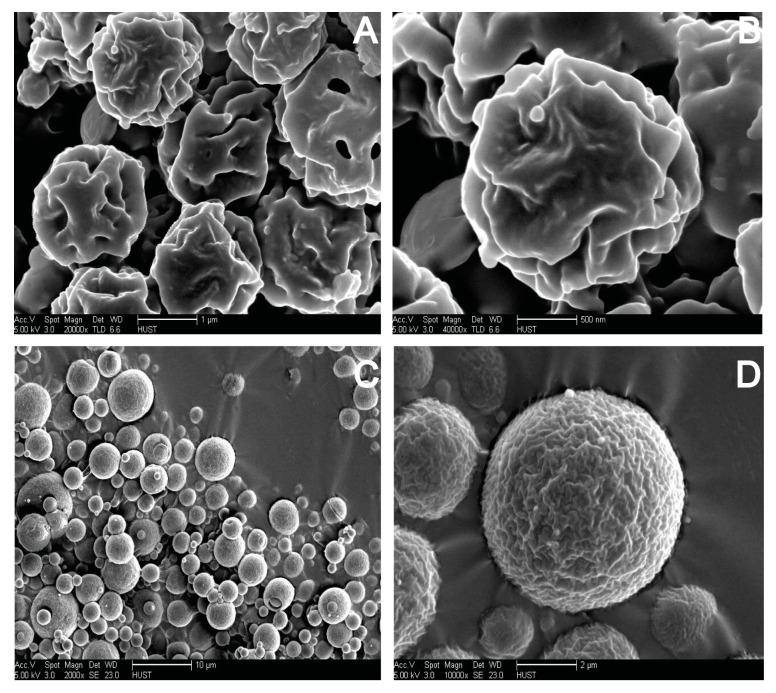
SEM images of S-SMEDDS maltodextrin (**A**: × 20000, bar = 1 μm; **B**: × 40000, bar = 500 nm), and acacia (**C**: × 2000, bar = 10 μm; **D**: × 10000, bar = 2 μm).

SEM observations of S-SMEDDS prepared with maltodextrin and acacia were presented in Figure 5. S-SMEDDS with PVP K30 was not observed because the reconstructed emulsions were as large as 400 nm, showing PVP K30 was not a suitable solid carrier. As shown in our previous study [20], the particles of S-SMEDDS prepared with dextran had a regular spherical shape with a particle size of 2–10 μm. There were slight dents on the surface of particles and the particles were well separated from each other. Figure 5A,B showed that the particles of S-SMEDDS prepared with maltodextrin were also substantially spherical, but the particle size was much smaller, about 1μm, and the dents on particle surface were much deeper than those of dextran. In addition, particles partially aggregated. The shape and size of S-SMEDDS prepared with acacia were similar to S-SMEDDS prepared with dextran, whose particles were unconventionally spherical shape with particle size between 2 and 10 μm. However, there were much more and denser pleats on the surface of S-SMEDDS of acacia (Figure 5C,D). The above results indicated that acacia could inhibit particles aggregation of S-SMEDDS just like dextran, which were more suitable as solid carriers for S-SMEDDS than maltodextrin.

Some studies [37,38] reported that maltodextrin had a stronger ability of resisting particles aggregation in spray-dried emulsions than lactose and low viscosity HPMC. However, different results were obtained in our studies, showing that there were obvious lipid leakages on the surface of S-SMEDDS with lactose as a carrier. For S-SMEDDS prepared with maltodextrin, the surface morphology of the particles was not good and particles were seriously aggregated. The different results may be due to more complex lipid components in SMEDDS than in dried emulsions.

**Figure 6:**
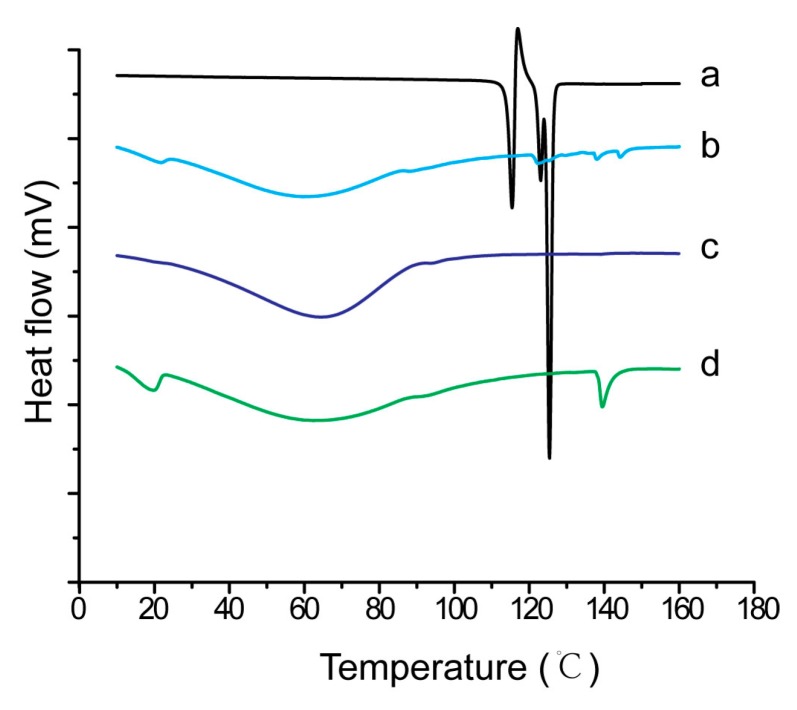
DSC curves of (**a**) pure nimodipine powder, (**b**) physical mixture of nimodipine and carriers, (**c**) S-SMEDDS of nimodipine with acacia and (**d**) S-SMEDDS of nimodipine with maltodextrin.

**Figure 7:**
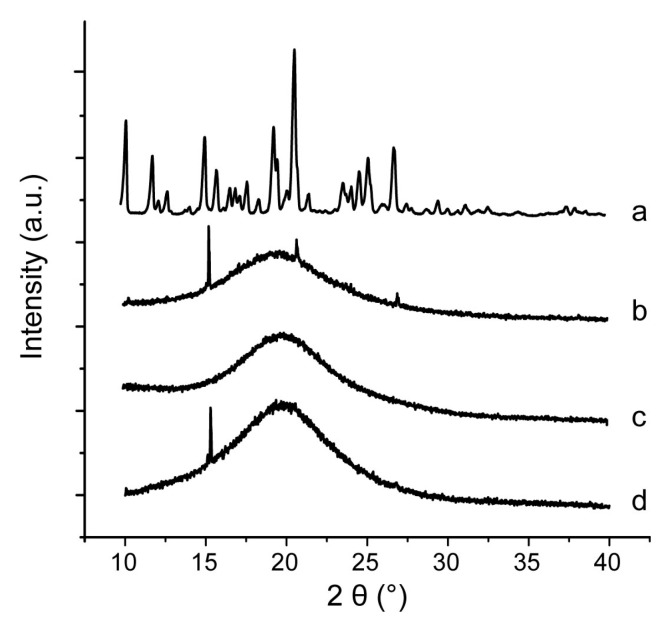
X-ray powder diffractometry of (**a**) pure nimodipine powder, (**b**) physical mixture of nimodipine and carriers, (**c**) S-SMEDDS of nimodipine with acacia and (**d**) S-SMEDDS of nimodipine with maltodextrin.

The DSC curves of nimodipine material, physical mixture of nimodipine and carriers, as well as S-SMEDDS-Ni were shown in Figure 6. There were three sharp endothermic peaks from 110 to 130 °C for nimodipine materia (Figure 6a) [39]. There were only two small endothermic peaks for the physical mixture because of the dilution of nimodipine by carriers (Figure 6b). Similarly, only a small number of diffraction peaks was observed for the physical mixture of nimodipine and carriers because of the dilution effect (Figure 7b). In our previous study [20], neither obvious endothermic peaks nor obvious diffraction peaks of nimodipine were observed in S-SMEDDS-Ni prepared with dextran. In this study, similar results were observed in S-SMEDDS-Ni of acacia (Figure 6c and Figure 7c), showing that acacia also inhibited crystallization of nimodipine in S-SMEDDS and nimodipine existed in an amorphous or molecular state in S-SMEDDS. By contrast, endothermic peaks or diffraction peaks of nimodipine were observed in S-SMEDDS of maltodextrin (Figure 6d and Figure 7d), indicating that maltodextrin had a poorer ability to inhibit crystallization than dextran and acacia.

The droplet sizes of reconstructed emulsions from S-SMEDDS determined by PCS were shown in Table 1. It had been proven that dextran did not affect the size of redispersed emulsion droplets of SMEDDS, which was about 44 nm and close to SMEDDS without any carriers [20]. However, the emulsion droplet sizes of redispersed S-SMEDDS prepared with maltodextrin, acacia and PVP K30 were all above 100 nm. Among them, S-SMEDDS of PVP K30 had the biggest increase in emulsion size, which was about 9.85 times that of SMEDDS. Polydispersity index of all redispersed S-SMEDDS increased compared with SMEDDS without any carriers.

In order to investigate relationships between the relative molecular mass of carriers and the in vitro properties of S-SMEDDS, S-SMEDDS with mannitol and lactose were also studied. When the relative molecular mass of the carrier was much smaller, such as mannitol and lactose, the lipid components were easier to leak out from the surface of the S-SMEDDS particles, and the particles were easier to aggregate. When the relative molecular mass of the carrier was relatively large, such as dextran 40 and acacia, the particles were separated well, and its effects of inhibiting crystallization of drugs was relatively strong. Further research should be required to confirm this phenomenon and clarify its mechanism.

### 3.5. Influences of Carriers on in Vitro Dissolution of S-SMEDDS

**Figure 8:**
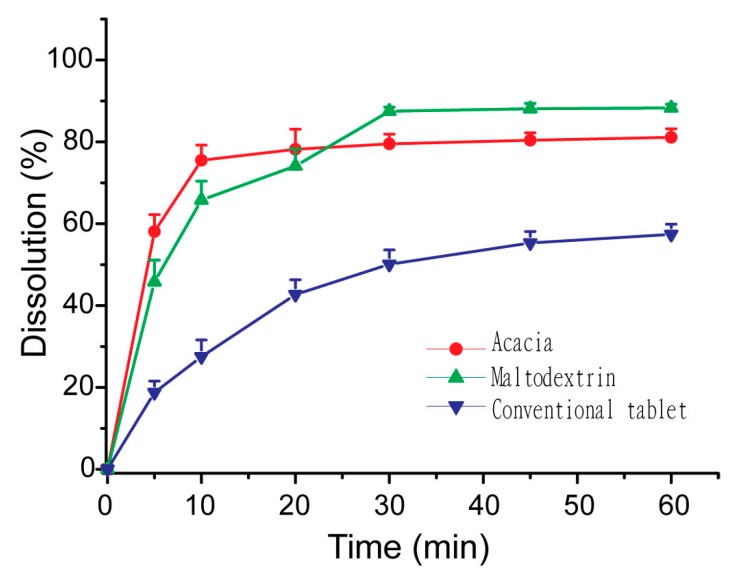
Dissolution profiles of nimodipine from S-SMEDDS with maltodextrin (▲), S-SMEDDS with acacia (⬤) and the conventional tablet (▼) in pH 4.5 acetate buffer containing 0.05% (*w*/*v*) of sodium dodecyl sulfate. Data were expressed as mean ± S.D. (*n* = 6).

As shown in Figure 8, S-SMEDDS prepared with maltodextrin and acacia had similar dissolution profiles to that of dextran [20]. They all released nimodipine quickly and completely, whose dissolution rates were much higher than that of commercially available tablets (*p* < 0.05). It indicated that S-SMEDDS with hydrophilic carriers could keep the improving effect of SMEDDS on dissolution of nimodipine *in vitro*.

## 4. Conclusions

The most critical issue in the development of S-SMEDDS is the selection of a suitable solid carrier to maintain the original advantages of SMEDDS. In this paper, the effects of hydrophilic excipients on the microstructural transitions of reconstructed microemulsions after being redispersed in water, drug loading, as well as precipitation and dissolution of S-SMEDDS in simulated gastric fluid were studied. It was found that hydrophilic excipients could delay the percolation transition of SMEDDS and enhance its drug-loading capacity. They also inhibited the precipitation of drugs when dispersed in simulated gastric liquid. The type of hydrophilic carriers had important influences on micromorphology, reconstruction of microemulsion and physical state of drugs in S-SMEDDS. The study provided systematic in vitro methods for screening carriers, whose results could also provide a basis for optimizing a hydrophilic carrier of S-SMEDDS.

## Figures and Tables

**Table 1:** Effects of hydrophilic carriers on in vitro properties of S-SMEDDS (*n* = 3, $\bar{x}$ ± SD).

Carrier	Relative Molecular Mass of Carrier	Droplet Size of Reconstructed Emulsion (nm)	Polydispersity Index of Reconstructed Emulsion	Lipid Leak of S-SMEDDS	Particle Separation of S-SMEDDS	Crystallization of S-SMEDDS
None (SMEDDS)	-	41.3 ± 5.7	0.13 ± 0.03	-	-	-
Mannitol	182	117.0 ± 7.2	0.16 ± 0.02	Yes	-	-
Lactose	342	124.0 ±10.6	0.14 ± 0.02	Yes	-	-
Maltodextrin	900–9000	139.5 ± 6.8	0.19 ± 0.05	No	Bad	Yes
Dextran 40 [20]	40,000	44.1 ± 4.7	0.25± 0.04	No	Good	No
PVP K30	50,000	407.5 ± 3.9	0.42 ± 0.09	No	-	-
Acacia	240,000–580,000	177.6 ± 14.6	0.41 ± 0.07	No	Good	No
